# *N*-[6-(Dibromo­meth­yl)-2-pyrid­yl]-2,2-dimethyl­propionamide

**DOI:** 10.1107/S1600536809007909

**Published:** 2009-03-11

**Authors:** Hoong-Kun Fun, Suchada Chantrapromma, Annada C. Maity, Rinku Chakrabarty, Shyamaprosad Goswami

**Affiliations:** a X-ray Crystallography Unit, School of Physics, Universiti Sains Malaysia, 11800 USM, Penang, Malaysia; b Crystal Materials Research Unit, Department of Chemistry, Faculty of Science, Prince of Songkla University, Hat-Yai, Songkhla 90112, Thailand; c Department of Chemistry, Bengal Engineering and Science University, Shibpur, Howrah 711 103, India

## Abstract

In the mol­ecular structure of the title compound, C_11_H_14_Br_2_N_2_O, the dimethyl­propionamide substituent is twisted slightly with respect to the pyridine ring, the inter­planar angle being 12.3 (2)°. The dibromo­methyl group is orientated in such a way that the two Br atoms are tilted away from the pyridine ring. In the crystal structure, mol­ecules are associated into supra­molecular chains by weak C—H⋯O inter­actions. The crystal is further stabilized by weak N—H⋯Br and C—H⋯N inter­actions.

## Related literature

For hydrogen-bond motifs, see: Bernstein *et al.* (1995 ▶). For mol­ecular recognition and *N*-bromo­succinimides, see, for example: Goswami & Mukherjee, (1997 ▶); Goswami *et al.* (2000 ▶, 2001 ▶, 2004 ▶). For the stability of the temperature controller used in the data collection, see: Cosier & Glazer (1986 ▶).
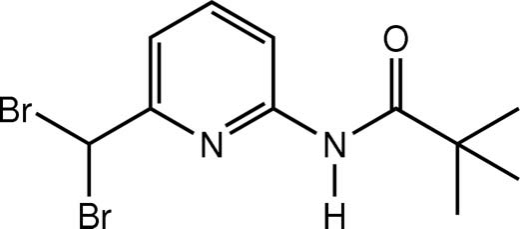


## Experimental

### 

#### Crystal data

C_11_H_14_Br_2_N_2_O
*M* *_r_* = 350.06
Monoclinic, 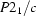

*a* = 13.2936 (7) Å
*b* = 8.4660 (3) Å
*c* = 11.9638 (6) Å
β = 99.195 (3)°
*V* = 1329.15 (11) Å^3^
*Z* = 4
Mo *K*α radiation
μ = 6.08 mm^−1^
*T* = 100 K
0.33 × 0.29 × 0.24 mm

#### Data collection

Bruker APEXII CCD area-detector diffractometer
Absorption correction: multi-scan (*SADABS*; Bruker, 2005 ▶) *T*_min_ = 0.114, *T*_max_ = 0.233
12087 measured reflections
3863 independent reflections
2954 reflections with *I* > 2σ(*I*)
*R*_int_ = 0.039

#### Refinement

*R*[*F* ^2^ > 2σ(*F* ^2^)] = 0.051
*wR*(*F* ^2^) = 0.153
*S* = 1.10
3863 reflections
152 parameters
H atoms treated by a mixture of independent and constrained refinement
Δρ_max_ = 1.98 e Å^−3^
Δρ_min_ = −1.13 e Å^−3^

### 

Data collection: *APEX2* (Bruker, 2005 ▶); cell refinement: *SAINT* (Bruker, 2005 ▶); data reduction: *SAINT*; program(s) used to solve structure: *SHELXTL* (Sheldrick, 2008 ▶); program(s) used to refine structure: *SHELXTL*; molecular graphics: *SHELXTL*; software used to prepare material for publication: *SHELXTL* and *PLATON* (Spek, 2009 ▶).

## Supplementary Material

Crystal structure: contains datablocks global, I. DOI: 10.1107/S1600536809007909/tk2385sup1.cif

Structure factors: contains datablocks I. DOI: 10.1107/S1600536809007909/tk2385Isup2.hkl

Additional supplementary materials:  crystallographic information; 3D view; checkCIF report

## Figures and Tables

**Table 1:** Hydrogen-bond geometry (Å, °)

*D*—H⋯*A*	*D*—H	H⋯*A*	*D*⋯*A*	*D*—H⋯*A*
N2—H1*N*2⋯Br1^i^	0.82 (5)	2.89 (4)	3.587 (4)	144 (4)
C3—H3*A*⋯O1^ii^	0.93	2.41	3.249 (5)	151
C4—H4*A*⋯O1	0.93	2.34	2.886 (5)	117
C9—H9*B*⋯N1^iii^	0.96	2.55	3.506 (6)	179

Symmetry codes: (i) 
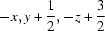
; (ii) 
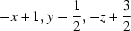
; (iii) 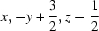.

**Fig. 1.**
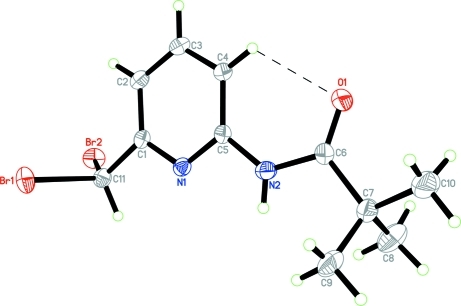
The molecular structure of (I), showing 50% probability displacement ellipsoids and the atomic numbering. The intramolecular C-H···O contact is drawn as a dashed line.

**Fig. 2.**
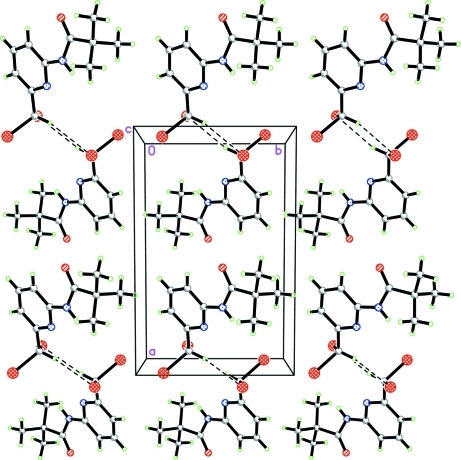
The crystal packing of the title compound, viewed down the c axis showing dimers with R22(6) motifs. Hydrogen bonds were drawn as dashed lines.

## supplementary crystallographic information

### Comment

Bromomethyl aromatic and heteroaromatic compounds (*e.g.* pyridine or naphthyridine derivatives) are important substrates and they have been used as the precursors for pharmacologically active compounds. Bromide compounds have applications in the synthesis of artificial receptors for molecular recognition research (Goswami & Mukherjee, 1997; Goswami *et al.*, 2000). We have also reported the *N*-bromosuccinimide reaction of various heterocycles in the absence or presence of water (Goswami *et al.*, 2001; 2004). We report here the crystal structure of the title compound which is a side-chain substituted with gem-dibromo moiety of pyridine.

In Fig. 1, the O1, N2, C6, C7 atoms lie on the same plane with the maximum deviation of 0.005 (5) Å being for atom C6. The mean plane through these atoms makes the dihedral angle of 12.3 (2)° with the mean plane through pyridine ring. This dihedral angle and the torsion angles C6–N2–C5–C4 = -14.4 (7)°, C5–N2–C6–O1 = 5.2 (7)° and C5–N2–C6–C7 = -174.0 (4)° indicate the orientation of the dimethylpropionamide substituent is slightly twisted with respect to the pyridine ring. The dibromomethyl group on the pyridine ring is orientated in such a way that the two bromine atoms are tilted away from the plane of pyridine ring. A weak intramolecular C4–H4A···O1 contact generates a S(6) ring motif (Bernstein *et al.*, 1995).

The crystal packing shows that the molecules are associated into supramolecular chains via weak C—H···O interactions (Table 1). The crystal is further stabilized by weak interactions of the type N—H···Br and C—H···N (Table 1).

### Experimental

To a 100 ml round bottom flask, a mixture of compound 1 (see Fig. 3) (3 g, 0.016 mol) and azobisisobutyronitrile (AIBN) (1.28 g, 7.79 mmol) were added. Dry CCl_4_ (30 ml) was added and the reaction mixture was heated to reflux for 30 min with vigorous stirring in the presence of light from a 60 W lamp. When all the materials were dissolved, *N*-bromosuccinimide (NBS) (2.78 g, 0.016 mol) was added slowly and reflux continued for 3 h. The reaction mixture was cooled, crushed ice added, and then extracted with CCl_4_ to afford the crude product. The brown liquid was purified by column chromatography over 100–200 mesh silica gel using 3% ethylacetate in petroleum ether (330–350 K) as eluent to yield a white dense liquid of compound 2 (Fig. 3) (2.12 g, yield 50%) and a crystalline solid 3 (2.18 g, yield 40%).

### Refinement

The amide-H atom was located in a difference map and refined isotropically; N-H = 0.82 (5)Å. The remaining H atoms were constrained in a riding motion approximation with d(C—H) = 0.93 Å and *U*_iso_=1.2*U*_eq_(C) for aromatic-H, d(C—H) = 0.98 Å and *U*_iso_=1.2*U*_eq_(C) for methine-H, and d(C—H) = 0.96 Å and *U*_iso_=1.5*U*_eq_(C) for methyl-H. A rotating group model was used for the methyl groups. The highest residual electron density peak was located at 0.86 Å from Br1 and the deepest hole was located at 0.86 Å from Br2.

### Crystal data

**Table:**

C_11_H_14_Br_2_N_2_O	*F*(000) = 688
*M**_r_* = 350.06	*D*_x_ = 1.749 Mg m^−^^3^
Monoclinic, *P*2_1_/*c*	Mo *K*α radiation, λ = 0.71073 Å
Hall symbol: -P 2ybc	Cell parameters from 3863 reflections
*a* = 13.2936 (7) Å	θ = 1.6–30.0°
*b* = 8.4660 (3) Å	µ = 6.08 mm^−^^1^
*c* = 11.9638 (6) Å	*T* = 100 K
β = 99.195 (3)°	Block, colorless
*V* = 1329.15 (11) Å^3^	0.33 × 0.29 × 0.24 mm
*Z* = 4	

### Data collection

**Table:**

Bruker APEXII CCD area-detector diffractometer	3863 independent reflections
Radiation source: sealed tube	2954 reflections with *I* > 2σ(*I*)
graphite	*R*_int_ = 0.039
φ and ω scans	θ_max_ = 30.0°, θ_min_ = 1.6°
Absorption correction: multi-scan (*SADABS*; Bruker, 2005)	*h* = −16→18
*T*_min_ = 0.114, *T*_max_ = 0.233	*k* = −9→11
12087 measured reflections	*l* = −16→16

### Refinement

**Table:**

Refinement on *F*^2^	Primary atom site location: structure-invariant direct methods
Least-squares matrix: full	Secondary atom site location: difference Fourier map
*R*[*F*^2^ > 2σ(*F*^2^)] = 0.051	Hydrogen site location: inferred from neighbouring sites
*wR*(*F*^2^) = 0.153	H atoms treated by a mixture of independent and constrained refinement
*S* = 1.10	*w* = 1/[σ^2^(*F*_o_^2^) + (0.0946*P*)^2^] where *P* = (*F*_o_^2^ + 2*F*_c_^2^)/3
3863 reflections	(Δ/σ)_max_ < 0.001
152 parameters	Δρ_max_ = 1.98 e Å^−^^3^
0 restraints	Δρ_min_ = −1.13 e Å^−^^3^

### Special details

**Table:**

Experimental. The crystal was placed in the cold stream of an Oxford Cryosystems Cobra open-flow nitrogen cryostat (Cosier & Glazer, 1986) operating at 100.0 (1) K.
Geometry. All e.s.d.'s (except the e.s.d. in the dihedral angle between two l.s. planes) are estimated using the full covariance matrix. The cell e.s.d.'s are taken into account individually in the estimation of e.s.d.'s in distances, angles and torsion angles; correlations between e.s.d.'s in cell parameters are only used when they are defined by crystal symmetry. An approximate (isotropic) treatment of cell e.s.d.'s is used for estimating e.s.d.'s involving l.s. planes.
Refinement. Refinement of *F*^2^ against ALL reflections. The weighted *R*-factor *wR* and goodness of fit *S* are based on *F*^2^, conventional *R*-factors *R* are based on *F*, with *F* set to zero for negative *F*^2^. The threshold expression of *F*^2^ > σ(*F*^2^) is used only for calculating *R*-factors(gt) *etc*. and is not relevant to the choice of reflections for refinement. *R*-factors based on *F*^2^ are statistically about twice as large as those based on *F*, and *R*- factors based on ALL data will be even larger.

### Fractional atomic coordinates and isotropic or equivalent isotropic displacement parameters (Å^2^)

**Table:**

	*x*	*y*	*z*	*U*_iso_*/*U*_eq_	
Br1	−0.01634 (3)	0.15918 (5)	0.82585 (4)	0.02738 (15)	
Br2	0.08741 (3)	0.31287 (5)	1.05799 (4)	0.02696 (15)	
O1	0.4376 (2)	0.5484 (4)	0.6250 (3)	0.0403 (9)	
N1	0.1855 (2)	0.4274 (4)	0.7746 (3)	0.0188 (6)	
N2	0.2728 (3)	0.5490 (4)	0.6511 (3)	0.0233 (7)	
C1	0.1747 (3)	0.3175 (4)	0.8522 (4)	0.0205 (8)	
C2	0.2499 (3)	0.2067 (5)	0.8910 (4)	0.0250 (9)	
H2A	0.2416	0.1345	0.9474	0.030*	
C3	0.3376 (3)	0.2089 (5)	0.8420 (4)	0.0269 (9)	
H3A	0.3887	0.1351	0.8643	0.032*	
C4	0.3500 (3)	0.3196 (4)	0.7602 (4)	0.0230 (8)	
H4A	0.4084	0.3216	0.7267	0.028*	
C5	0.2719 (3)	0.4275 (5)	0.7302 (3)	0.0191 (7)	
C6	0.3540 (3)	0.6069 (5)	0.6056 (4)	0.0237 (8)	
C7	0.3295 (3)	0.7544 (5)	0.5291 (4)	0.0278 (9)	
C8	0.3325 (5)	0.8986 (6)	0.6075 (5)	0.0463 (13)	
H8A	0.3962	0.9001	0.6584	0.070*	
H8B	0.2774	0.8923	0.6502	0.070*	
H8C	0.3260	0.9933	0.5629	0.070*	
C9	0.2253 (4)	0.7427 (6)	0.4534 (5)	0.0441 (14)	
H9A	0.2227	0.6484	0.4084	0.066*	
H9B	0.2152	0.8332	0.4045	0.066*	
H9C	0.1727	0.7391	0.4998	0.066*	
C10	0.4117 (4)	0.7706 (7)	0.4552 (5)	0.0452 (14)	
H10A	0.4150	0.6753	0.4124	0.068*	
H10B	0.4763	0.7887	0.5022	0.068*	
H10C	0.3959	0.8579	0.4043	0.068*	
C11	0.0748 (3)	0.3265 (4)	0.8950 (3)	0.0212 (8)	
H11A	0.0433	0.4283	0.8713	0.025*	
H1N2	0.224 (3)	0.609 (6)	0.638 (4)	0.023 (12)*	

### Atomic displacement parameters (Å^2^)

**Table:**

	*U*^11^	*U*^22^	*U*^33^	*U*^12^	*U*^13^	*U*^23^
Br1	0.0269 (2)	0.0317 (3)	0.0252 (2)	−0.00904 (17)	0.00912 (17)	−0.00740 (16)
Br2	0.0314 (3)	0.0321 (3)	0.0176 (2)	0.00285 (17)	0.00477 (17)	0.00017 (15)
O1	0.0224 (16)	0.0365 (19)	0.064 (3)	0.0035 (14)	0.0128 (16)	0.0234 (17)
N1	0.0202 (16)	0.0166 (15)	0.0189 (15)	−0.0010 (12)	0.0012 (13)	−0.0006 (12)
N2	0.0215 (17)	0.0221 (17)	0.0271 (18)	0.0045 (14)	0.0064 (14)	0.0044 (14)
C1	0.0213 (19)	0.0155 (18)	0.025 (2)	−0.0025 (14)	0.0030 (16)	−0.0011 (14)
C2	0.027 (2)	0.0176 (18)	0.031 (2)	0.0014 (15)	0.0064 (17)	0.0041 (16)
C3	0.026 (2)	0.0172 (19)	0.037 (2)	0.0033 (16)	0.0029 (18)	0.0031 (17)
C4	0.0197 (19)	0.0169 (19)	0.033 (2)	0.0014 (14)	0.0070 (17)	0.0001 (15)
C5	0.0186 (18)	0.0168 (18)	0.0219 (18)	−0.0021 (14)	0.0031 (15)	−0.0005 (14)
C6	0.0211 (19)	0.0207 (19)	0.030 (2)	−0.0017 (15)	0.0057 (16)	0.0011 (16)
C7	0.027 (2)	0.024 (2)	0.034 (2)	0.0004 (17)	0.0103 (18)	0.0048 (17)
C8	0.065 (4)	0.022 (2)	0.053 (3)	0.003 (2)	0.010 (3)	0.000 (2)
C9	0.043 (3)	0.035 (3)	0.051 (3)	−0.003 (2)	−0.004 (2)	0.025 (2)
C10	0.046 (3)	0.043 (3)	0.052 (3)	0.012 (2)	0.023 (3)	0.022 (3)
C11	0.024 (2)	0.0194 (19)	0.0206 (19)	−0.0010 (15)	0.0044 (15)	−0.0021 (14)

### Geometric parameters (Å, °)

**Table:**

Br1—C11	1.959 (4)	C4—H4A	0.9300
Br2—C11	1.934 (4)	C6—C7	1.551 (6)
O1—C6	1.205 (5)	C7—C10	1.518 (6)
N1—C1	1.338 (5)	C7—C9	1.532 (7)
N1—C5	1.341 (5)	C7—C8	1.536 (7)
N2—C6	1.374 (5)	C8—H8A	0.9600
N2—C5	1.400 (5)	C8—H8B	0.9600
N2—H1N2	0.82 (5)	C8—H8C	0.9600
C1—C2	1.395 (6)	C9—H9A	0.9600
C1—C11	1.500 (6)	C9—H9B	0.9600
C2—C3	1.386 (6)	C9—H9C	0.9600
C2—H2A	0.9300	C10—H10A	0.9600
C3—C4	1.384 (6)	C10—H10B	0.9600
C3—H3A	0.9300	C10—H10C	0.9600
C4—C5	1.385 (5)	C11—H11A	0.9800
			
C1—N1—C5	117.9 (3)	C9—C7—C6	112.4 (4)
C6—N2—C5	128.5 (4)	C8—C7—C6	107.2 (4)
C6—N2—H1N2	110 (3)	C7—C8—H8A	109.5
C5—N2—H1N2	120 (3)	C7—C8—H8B	109.5
N1—C1—C2	123.2 (4)	H8A—C8—H8B	109.5
N1—C1—C11	113.6 (3)	C7—C8—H8C	109.5
C2—C1—C11	123.2 (4)	H8A—C8—H8C	109.5
C3—C2—C1	117.2 (4)	H8B—C8—H8C	109.5
C3—C2—H2A	121.4	C7—C9—H9A	109.5
C1—C2—H2A	121.4	C7—C9—H9B	109.5
C4—C3—C2	120.7 (4)	H9A—C9—H9B	109.5
C4—C3—H3A	119.6	C7—C9—H9C	109.5
C2—C3—H3A	119.6	H9A—C9—H9C	109.5
C3—C4—C5	117.5 (4)	H9B—C9—H9C	109.5
C3—C4—H4A	121.3	C7—C10—H10A	109.5
C5—C4—H4A	121.3	C7—C10—H10B	109.5
N1—C5—C4	123.4 (4)	H10A—C10—H10B	109.5
N1—C5—N2	111.6 (3)	C7—C10—H10C	109.5
C4—C5—N2	124.9 (4)	H10A—C10—H10C	109.5
O1—C6—N2	122.4 (4)	H10B—C10—H10C	109.5
O1—C6—C7	123.0 (4)	C1—C11—Br2	113.7 (3)
N2—C6—C7	114.6 (3)	C1—C11—Br1	110.0 (3)
C10—C7—C9	109.2 (4)	Br2—C11—Br1	109.32 (19)
C10—C7—C8	109.4 (4)	C1—C11—H11A	107.9
C9—C7—C8	110.2 (4)	Br2—C11—H11A	107.9
C10—C7—C6	108.2 (4)	Br1—C11—H11A	107.9
			
C5—N1—C1—C2	1.9 (6)	C5—N2—C6—O1	5.2 (7)
C5—N1—C1—C11	−179.1 (3)	C5—N2—C6—C7	−174.0 (4)
N1—C1—C2—C3	−2.8 (6)	O1—C6—C7—C10	19.8 (6)
C11—C1—C2—C3	178.3 (4)	N2—C6—C7—C10	−161.0 (4)
C1—C2—C3—C4	1.6 (7)	O1—C6—C7—C9	140.5 (5)
C2—C3—C4—C5	0.3 (7)	N2—C6—C7—C9	−40.3 (6)
C1—N1—C5—C4	0.3 (6)	O1—C6—C7—C8	−98.2 (5)
C1—N1—C5—N2	−179.7 (3)	N2—C6—C7—C8	81.0 (5)
C3—C4—C5—N1	−1.3 (6)	N1—C1—C11—Br2	−133.6 (3)
C3—C4—C5—N2	178.6 (4)	C2—C1—C11—Br2	45.5 (5)
C6—N2—C5—N1	165.5 (4)	N1—C1—C11—Br1	103.5 (3)
C6—N2—C5—C4	−14.4 (7)	C2—C1—C11—Br1	−77.5 (5)

### Hydrogen-bond geometry (Å, °)

**Table:**

*D*—H···*A*	*D*—H	H···*A*	*D*···*A*	*D*—H···*A*
N2—H1N2···Br1^i^	0.82 (5)	2.89 (4)	3.587 (4)	144 (4)
C3—H3A···O1^ii^	0.93	2.41	3.249 (5)	151
C4—H4A···O1	0.93	2.34	2.886 (5)	117
C9—H9B···N1^iii^	0.96	2.55	3.506 (6)	179

Symmetry codes: (i) −*x*, *y*+1/2, −*z*+3/2; (ii) −*x*+1, *y*−1/2, −*z*+3/2;  (iii) *x*, −*y*+3/2, *z*−1/2.
